# CBD in the Treatment of Epilepsy

**DOI:** 10.3390/molecules29091981

**Published:** 2024-04-25

**Authors:** Kinga Borowicz-Reutt, Julia Czernia, Marlena Krawczyk

**Affiliations:** Independent Unit of Experimental Neuropathophysiology, Department of Toxicology, Medical University of Lublin, Jaczewskiego 8b, PL-20-090 Lublin, Poland; julia.czernia@umlub.pl (J.C.); marlena.krawczyk1@umlub.pl (M.K.)

**Keywords:** phytocannabinoids, CBD, seizure, epilepsy, efficacy, safety

## Abstract

It has been several years since highly purified cannabidiol (CBD) was registered as a medication that can be used in children of at least 2 years of age to treat different types of seizures related to Lennox–Gastaut syndrome (LGS), Dravet syndrome (DS), and more recently tuberous sclerosis complex (TSC). During this time, 39 randomized clinical trials (RCTs) and 13 meta-analyses on the efficacy and safety of CBD treatment have been published. Each of the meta-analyses had its own criteria for the RCTs’ inclusion and, therefore, slightly different interpretations of the analyzed data. Each of them contributed in its own way to the understanding of CBD pharmacology, mechanisms of therapeutic action, development of adverse reactions, and drug–drug interactions. Hence, it seemed reasonable to gather the most relevant data in one article and present all the current knowledge on the use of CBD in epilepsy. The results of the 13 meta-analyses presented herein confirmed the effectiveness and safety of CBD in children and adolescents with DREs. In adults, reliable conclusions cannot be drawn due to insufficient data.

## 1. Introduction

**Figure 1:**
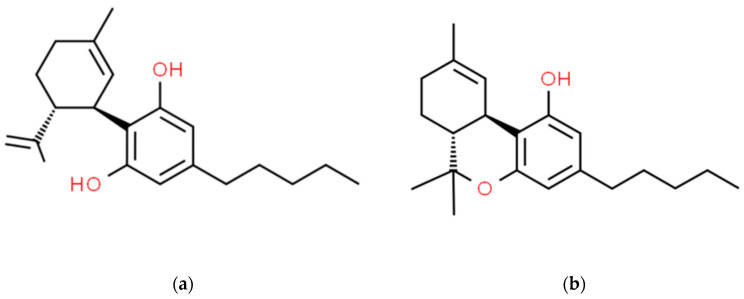
Chemical structures of CBD (**a**) and THC (**b**).

Despite the availability of various types of antiepileptic therapies, including pharmacological therapy, deep brain stimulation, and surgery, the percentage of patients with treatment-resistant epilepsy remains stable. Cannabis derived from the *Cannabis sativa* plant, also commonly referred to as marijuana (obtained from fermented female inflorescences), has been used since antiquity for recreational, spiritual, or medical purposes. Currently, the leading properties used in medicine are anticonvulsant and analgesic ones. Most of the over 400 active substances of *Cannabis sativa* are terpenes, sesquiterpenes, and cannabinoids. Two prominent phytocannabinoids found in the plant resin are cannabidiol (CBD) and delta-9-tetrahydrocannabinol (THC) (Figure 1). So far, numerous studies have demonstrated that cannabinoids may affect various aspects of neurological diseases, such as sleep disorders, stress consequences, depression, anxiety, autism spectrum disorder, and attention deficit hyperactivity disorder (ADHD) [1,2,3]. In contrast to THC, CBD lacks psychoactive properties, does not produce euphoric or other intrusive side effects, and usually does not pose a risk regarding abuse or dependence development. While CBD has been reported to exhibit clear-cut antiseizure properties, contradictory pro- and anticonvulsant effects have been observed for THC. The advantageous anticonvulsant efficacy confirmed in numerous studies and their meta-analyses prompted the US Food and Drug Administration (FDA) and European Medicines Agency (EMA) to approve a highly purified CBD formulation for the supportive treatment of seizures related to Dravet syndrome (DS), Lennox–Gastaut syndrome (LGS), and tuberous sclerosis complex (TSC) in patients at least 2 years old [4,5,6,7,8,9,10,11,12].

Both exogenous (from *Cannabis sativa*) and endogenous cannabinoids, their metabolizing enzymes, like fatty acid amide hydrolase, and cannabinoid receptors form the endocannabinoid system (ECS). The ECS is involved in the regulation of neurotransmission and neural development, brain metabolism and energy balance, stress and emotions, as well as the inhibition of the inflammatory processes and mechanisms of addiction. The two most-studied endocannabinoid ligands are anandamide (ANA), known also as N-arachidonoyl-ethanolamine (NAE), and 2-arachidonoylglycerol (2-AG). Interestingly, “ananda” in Sanskrit means absolute happiness. Endocannabinoids are considered as atypical messengers participating in the retrograde signaling mechanism, i.e., from postsynaptic to presynaptic terminals. They are synthesized on demand from membrane phospholipids. ANA is created by the activation of either the Ca^2+^-dependent or Ca^2+^-independent N-acyltransferase pathways. In the case of 2-AG, two-step biosynthesis takes place. At first, 1,2-diacylglycerol (DAG) is formed by phospholipase C. Then, DAG is esterified by diacylglycerol lipase (DAGL) to 2-AG. The endocannabinoids released to the synaptic cleft bind to their specific target points. The best-known receptors belonging to the ECS, cannabinoid type-1 and type-2 receptors (CB1R and CB2R, respectively), are located throughout the central and peripheral nervous system. CB1Rs can mostly be found in the central nervous system, including the hippocampus, cerebral cortex, cerebellum, hypothalamus, and amygdala. They are also located in the cardiovascular system, lungs, gastrointestinal tract, and kidneys. CB2Rs are predominant in the peripheral nervous system, immune system, and hematopoietic cells. Cannabinoids might also act through the vanilloid receptors (TRPV1, TRPV2, TRPV3, TRPV4, TRPA1, and TRPM8) and metabotropic receptors, such as GPR55, GPR3, GPR6, GPR12, and GPR19. Compared to CBD, THC has significantly higher affinity to CB1Rs and CB2Rs. After separation from the receptors, endocannabinoids are reuptaken to the presynaptic terminals by membrane transporters and inactivated by specific enzymes. ANA is predominantly degraded by the fatty acid amide hydroxylase enzyme (FAAH), while 2-AG is metabolized by the monoacylglycerol lipase (MAGL). In both cases, the two final products are arachidonic acid and glycerol [4,13,14,15,16].

## 2. CBD Mechanisms of Action

**Figure 2:**
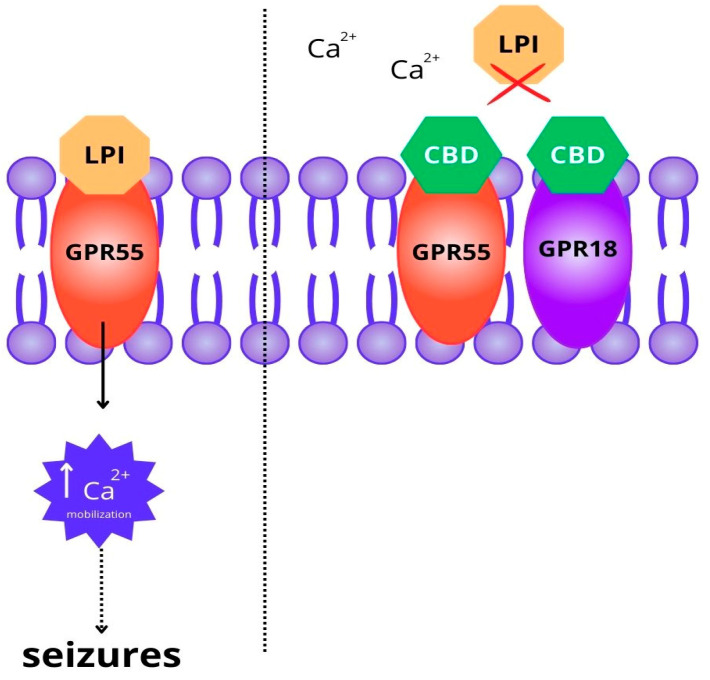
Scheme of main mechanisms of action of CBD through GPR receptors in preventing seizure outcomes in epilepsy. Lysophosphatidylinositol (LPI) is an endogenous endocannabinoid neurotransmitter that activates the GPR55 receptor. This activation leads to an increase in intracellular calcium mobilization and the development of seizures. CBD (cannabidiol) acts as an antagonist of the GPR55 and GPR18 receptors, blocking the effects of LPI and reducing neuronal excitability.

The exact mechanisms of the multiple therapeutic actions of CBD remain unclear. This cannabinoid was described as an inverse agonist and negative allosteric modulator of the G-protein-coupled seven-transmembrane CB1 and CB2 receptors. Additionally, CBD may also be a partial agonist of the CB2 receptor. Through the direct influence on the ECS, CBD may reduce pain, distress, inflammatory processes, oxidative stress, and neuronal excitability degeneration, and it maintains glial cell homeostasis, as well as inhibits the development of cancer and neurodegenerative diseases. However, at clinical concentrations, CBD showed low affinity for the cannabinoid receptors, although it may still act as a negative allosteric modulator at the CB1 receptor. Therefore, it is not certain to what extent the anticonvulsant effect of phytocannabinoids is due to their action on the CB receptors. Among the other considered mechanisms that can mediate the antiseizure properties of CBD, there is the antagonistic action of CBD on G-protein orphan receptors, such as GPR18 and GPR55 (see Figure 2), and the transient receptor potential cation channel subfamily members (vanilloid receptors), like TRPA1 and TRPV1-4. CBD is also an inverse agonist of the GPR3, GPR6, and GPR 12 receptors, as well as an antagonist of the µ and δ opioid receptors. Presently, the antiseizure properties of CBD are thought to be mainly the result of the modulation of the intracellular Ca2+ levels by blocking the T-type voltage-gated calcium channels, the antagonistic action on the GPR55 receptor, as well as the full agonistic action on the vanilloid TRPA1 and TRPV1-4 channels. Additionally, the inhibition of adenosine reuptake, positive allosteric modulation of GABA_A_ and glycine receptors, positive allosteric modulation of 5-HT1A-, and partially agonistic action on 5-HT2A-mediated neurotransmission, antagonistic and negative allosteric modulation of 5HT3 channels and α7 nicotinic receptors, inhibition of the currents conducted through the voltage-gated Cav3.1, Cav3.2, and Cav3.3 calcium channels, and reducing the synthesis of inflammatory cytokines through the agonistic action on peroxisome proliferator-activated receptor-gamma (PPARγ) are also taken into account. To date, it has not been determined whether exogenous phytocannabinoids affect the production or function of natural endocannabinoids ANA and 2-AG [6,7,17,18,19,20,21,22].

The role of the GPR55 receptor in seizure processes was confirmed in several studies. GPR55 is activated by certain cannabinoids and lysophosphatidylinositol (LPI), an endogenous endocannabinoid neurotransmitter. CBD, being an antagonist of this receptor, blocks the effects of LPI. One of the functions of LPI is increasing the hippocampal excitatory–inhibitory ratio through increased intracellular calcium mobilization. Acute seizures upregulate GPR55 and increase the LPI concentrations, forming a harmful positive feedback loop. CBD restores the balance between excitation and inhibition, thus reducing the neuronal excitability and the occurrence of repeated seizures. The role of GPR55 in reducing PTZ seizures was also confirmed in an experimental zebrafish model. The CBD-induced blockade of GPR55 led to significant seizure relief, but the convulsions were not entirely abolished. When both GPR55 and CB1 were blocked, the reduction in seizures was insignificantly greater, so CB1 receptors may play a minor role in the anticonvulsant action of CBD. The same applies to GPR18 receptors. CBD blocks these receptors, thus decreasing the intracellular Ca^2+^ mobilization. N-methyl-D-aspartate (NMDA) is recognized as a chemoconvulsant, and the activation of the NMDA receptors leads to seizures and other neurological disorders. Furthermore, in mouse models of NMDA-induced seizures, CBD showed antiseizure effects, suggesting the role of the NMDA receptors [4,23,24,25].

In addition to CBD cannabinoids, endocannabinoid ligands are generally considered to inhibit seizures; however, the available data are contradictory. While ANA showed some anticonvulsant effects, the loss of the FAAH enzyme catabolizing ANA led to proconvulsive action [4,19].

## 3. Pharmacokinetic Interactions between CBD and Clobazam and Stiripentol

**Figure 3:**
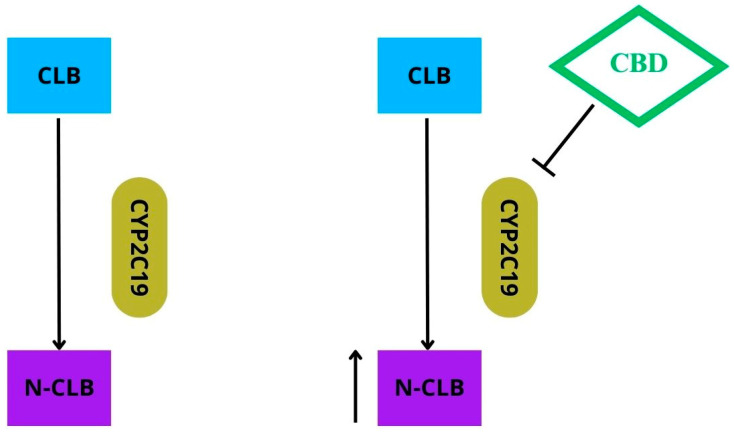
Clobazam (CLB) is biotransformed into active metabolite N-desmethylclobazam (N-CLB) via cytochrome CYP2C19. CBD inhibits the function of the cytochrome CYP2C19; thus, concentration of N-CLB increases.

In clinical conditions, CBD, in accordance with the FDA and EMA approvals, is used as an add-on therapy with other antiepileptic medications. In children with LGS and DS, the most effective and commonly used antiseizure drug seems to be clobazam. Noteworthily, CBD proved to be more effective when combined with this drug. The interactions between the two active substances appear to be primarily pharmacokinetic. CBD inhibits the function of the cytochrome CYP 2C19 and thus also the metabolism of clobazam, leading to around a three-fold increase in the plasma concentrations of N-desmethylclobazam (N-CLB), the active metabolite of clobazam (see Figure 3).

In turn, clobazam decreases the function of CYP2D6 and raises the plasma levels of 7-hydroxy-CBD, the active metabolite of CBD, by around 1.5 times. Interestingly, the N-CLB levels did not increase in those patients treated previously with stiripentol, a potent CYP2C19 inhibitor. This indicates that stiripentol has managed to saturate the binding sites within this cytochrome, which cannot be further inhibited by CBD. In patients taking stiripentol, a seizure frequency reduction (SFR) occurred in 80% and 50% of the participants treated with CBD and a placebo, respectively. It can be assumed that the rise in N-CLB levels is not entirely responsible for the antiseizure activity of CBD. Therefore, the possibility of a pharmacodynamic synergistic interaction between CBD and clobazam cannot be excluded [6,7,10].

## 4. Efficacy of CBD in Epilepsy Treatment

The available meta-analyses indicate that CBD is the most effective cannabinoid reducing the seizure frequency (SF) in both experimental and clinical conditions. Most of the randomized clinical trials (RCTs) were conducted in children and adolescents with LGS, DS, and TSC, the most severe forms of drug-resistant epilepsy (DRE) in these age groups [6,12,20,26].

Approximately two-thirds of the patients with DS carry loss-of-function mutations in the voltage-gated sodium channel α1 subunit gene (*SCN1A*). The symptomatology of DS includes convulsive seizures, like tonic, atonic, clonic, and tonic–clonic seizures. Within them, drop seizures can be distinguished, i.e., atonic, tonic, and tonic–clonic convulsions. Patients with DS also present non-convulsive seizures, such as absences. Similarly, the multiple seizure types associated with LGS include drop seizures (tonic, atonic, or tonic–atonic), atypical absences, and other convulsive seizures (clonic, tonic–clonic, etc.). In TSC, early-onset epilepsy begins with focal seizures that can precede, coexist with, or evolve into infantile spasms. An infantile spasm is a serious condition that usually occurs in the first year of life. In this disorder, the body of a child suddenly leans forward or backward and then stiffens. Nevertheless, patients with TSC can experience almost all types of convulsions, such as tonic, atonic, or tonic–clonic convulsions [7,27].

Below, we will present ten meta-analyses of RCTs and two systematic reviews dealing with the efficacy and safety profiles of CBD used as an add-on therapy in patients with various DREs. All the studies evaluated a total of 225 RCTs involving 17.400 subjects. Three meta-analyses were carried out in children and adolescents [6,7,8], while the remaining ones were additionally in adults [9,20,26,28,29,30]. However, adults made up the lowest percentage of the patients studied. In the majority of the meta-analyses, CBD was used at doses of 10 and 20 mg/kg/day [6,7,8,12,26,28]; however, the cannabinoid was also administered at higher doses of 25 and 50 mg/kg/day [9] or at dose ranges varying from 2.5 to 60 mg/kg/day [20,29,30]. Almost all the publications taken into consideration dealt with patients with DS and/or LGS [6,7,8,26,28,30]; some others also comprised TSC sufferers [9,12,20], and one of them covered the widest spectrum of patients, including those with 1. CDKL5 deficiency disorder, 2. SYNGAP1 encephalopathy, 3. epilepsy with myoclonic absences, as well as 4. Aicardi, 5. Dup15q, and 6. Doose syndromes, all of them being the common causes of epileptic encephalopathies [9].

The common observation of all the meta-analyses was that CBD significantly and dose-dependently reduced the intensity and frequency of seizures and incidence of status epilepticus accompanying epilepsy encephalopathies. It was reflected by remarkable reductions in the total and ≥50% SFR parameters. The level of significance varied from *p* < 0.01 to *p* < 0.001 for the CBD treatment without clobazam and was established as *p* < 0.0001 for the combined therapy with CBZ and clobazam [6,7,8,28]. Simultaneously, this cannabinoid, also dose-dependently, increased the incidence, intensity, and frequency of treatment-related adverse effects, mostly diarrhea, somnolence, and sedation. The withdrawal rates in the patients treated with CBD and antiepileptic drugs were similar or even lower than those only administered antiepileptics. Nevertheless, a direct comparison of the results was impossible because different statistical methods were used in the individual meta-analyses, i.e., the differences between trial outcomes were presented in the form of risk ratios [6,7,8,9], odds ratios [20,28,30], or standardized mean differences [29]. In one case, the authors did not calculate the level of significance but instead set the level of evidence quality [12].

Four meta-analyses compared the effect of CBD (at doses of 10 and 20 mg/kg/day) with and without the co-administration of clobazam in DS and/or LGS patients. All of them showed that the CBD treatment provided a higher rate of SFR in comparison to a placebo both in the patients taking and not taking this benzodiazepine. Moreover, the groups co-treated with CBD and CLO presented significantly greater antiseizure effects when compared to CBD monotherapy. The pharmacological studies revealed that the antiseizure effect of CBD is not only dependent on the increased brain concentrations of clobazam. Also, a synergistic interaction between CBD and clobazam may be probable. Noteworthily, the patients in the clobazam-off group were recruited from those whose previous clobazam therapy was ineffective. Therefore, the participants not treated with this antiepileptic can be considered as exceptionally difficult to treat. On the other hand, the treatment with CBD, when compared to a placebo, was related to a higher rate of adverse effects, primarily somnolence, sedation, rash, pneumonia, or aggression. Most of them were, however, mild to moderate in intensity [7,8,26,28].

What we consider important in individual meta-analyses and systematic reviews is as follows. One of the conclusions drawn by Lattanzi et al. [6] was that LGS or DS patients who tolerate CBD at 10 mg/kg but require further SFR should consider titrating the dose up to the maximum recommended 20 mg/kg. It is also noteworthy that the patients with LGS treated with adjunctive CBD achieved better control of both convulsive and non-convulsive seizures. However, in patients with DS, CBD did not provide a greater reduction in non-convulsive seizures when compared to a placebo. This finding can be explained by the greater activity of CBD against convulsive seizures, or the size of the group was too small to detect the difference between the CBD and placebo arms. It is also possible that the non-convulsive seizures in the developmentally delayed children with DS were not counted correctly by the caregivers. Despite the lack of a significant effect, a positive dose–response relationship was observed in the non-convulsive seizures since the CBD at the higher dose (20 mg/kg) provided greater antiseizure effects when compared to the dose of 10 mg/kg. Additionally, a significantly higher percentage of patients or caregivers observed an improvement in the general condition of the patients treated with CBD when compared to the placebo group [6].

Lattanzi et al. [7] confirmed the CBD efficacy mostly against DS-related convulsive seizures. Importantly, an improvement was observed in patients who did not respond to treatment with several antiepileptic drugs, with a median of four medications. A greater effect of treatment was found in the clobazam-on compared with the clobazam-off patients [7].

Similarly, a study by Devinsky et al. [28] revealed that, in the LGS cohort not treated with clobazam, an increased number of patients on CBD (20 mg/kg/day) experienced an increase in seizure frequency compared to a placebo. This was not observed for the patients taking clobazam. In the patients co-administered CBD, clobazam, and stiripentol (i.e., with the N-CLB levels maximally elevated by stiripentol), both the SFR and 50% responder rate favored the CBD treatment vs. a placebo [28].

Lattanzi et al. [9] conducted a systematic review in which they showed the efficacy and safety of adjunct CBD in patients with TSC-related 1. focal motor seizures without impairment of awareness, 2. focal seizures with impairment of awareness, 3. focal seizures evolving to bilateral motor seizures, and 4. generalized seizures (tonic–clonic, tonic, clonic, or atonic) compared to placebo groups. The 50% responder rate in the CBD-treated patients was around 40%. In addition, CBD improved the electroencephalographic image, markedly reducing the seizure discharges. Furthermore, the effectiveness of CBD add-on treatment was observed in single pediatric and adult patients with rare epilepsy syndromes, including (1) CDKL5 deficiency disorder, (2) SYNGAP1 encephalopathy, (3) epilepsy with myoclonic absences, as well as (4) Aicardi, (5) Dup15q, and (6) Doose syndromes, all of them being common causes of epileptic encephalopathies. The long-term response to the treatment was observed over 2–4 years in the patients with CDKL5 epileptic encephalopathy and epilepsy with myoclonic absences. Also, a significant SFR of 80–95% and the normalization of the encephalogram were observed in patients with SYNGAP1 developmental and epileptic encephalopathy treated with adjunctive CBD. Furthermore, a CBD add-on treatment exhibited a relevant SFR in patients with (1) Sturge–Weber syndrome, (2) focal cortical dysplasia, (3) lissencephaly, (4) brain tumor-related epilepsy, (5) frontal and temporal lobe epilepsy, and (6) febrile-related epilepsy. In addition to the antiseizure effect, improvements in cognition, behavior, mood, and many quality of life areas were reported in CBD-treated patients with TSC and other types of DRE. These effects were independent of the seizure reduction [9].

The two next meta-analyses below confirmed the beneficial effects and safety of the adjunctive CBD in patients with LGS, DS, and TSC. Interestingly, this treatment improved the perception of the clinical condition of the patients by 21% [12]. CBD proved to be most effective in patients with LGS. The recommended daily dose range is 5–20 mg/kg administered orally in two equally divided doses [20].

Bilbao and Spanagel [29] systematically reviewed trials evaluating the effect of different medical cannabinoid products (CBD, dronabinol, nabilone, cannabidiol, and nabiximols) on a variety of disorders, including epilepsy. Only CBD showed significant antiseizure efficacy. In addition, this medication moderately reduced the symptoms of parkinsonism [29].

Devi et al. [30] compared the short- and long-term effectiveness and safety of six antiseizure medications (CBD, clobazam, felbamate, lamotrigine, rufinamide, and topiramate) used in the treatment of LGS. Clobazam (1 mg/kg/day) achieved the highest place in the hierarchy of drug efficiency. Furthermore, the long-term treatment with clobazam resulted in a significantly higher proportion of patients (78%) with reductions in drop-seizures, while the long-term use of CBD (20 mg/kg/day) was associated with a higher frequency of adverse effects (96%). The authors concluded that clobazam, CBD, and rufinamide are the most effective and safest in terms of both the short- and long-term outcomes, followed by topiramate, lamotrigine, and low-dose clobazam (0.25 mg/kg/day) [30].

A brief summary of the data from the main meta-analyses is presented in Table 1.

## 5. Non-Medical Cannabis Use

Also, so-called recreational marijuana is used to treat seizures. It differs from medical marijuana in that it does not contain standardized CBD/THC proportions. However, the number of people using these products to reduce seizures is difficult to estimate because marijuana can be of various origins [31]. In a scoping review, Li et al. [11] considered the application of non-medical cannabis (NMC) in people with epilepsy. The factors positively correlated with NMC use in such patients were male sex, younger adult age, lower education and socio-economic status, psychiatric comorbidities, and being single. According to the literature, children with epilepsy took NMC primarily for seizure control, with an oral route of administration and high CBD/THC ratios. Adults with epilepsy used NMC with different CBD/THC proportions and for various reasons, including recreation, predominantly through smoking. Patients are faithful to the concept that NMC is more natural and safer than pharmacotherapy. Nevertheless, NMC (with often relevant THC content) has not been clearly proven to have an antiseizure effect. Simultaneously, it may produce various harmful effects. Most of the studies reported a lifetime prevalence of NMC use in people with epilepsy between 10 and 40%. Unfortunately, the unbeneficial effects of NMC and addiction rates were not evaluated in formal neuropsychological tests. As mentioned before, THC has greater affinity to the CB1 and CB2 receptors than CBD, through which it produces psychoactive symptoms [32]. However, depending on the route of admission, marijuana with THC may induce other adverse effects. For instance, smoking cannabis may lead to severe lung disorders such as bullous lung disease, hypersensitivity pneumonitis, and lung cancer [33].

## 6. Adverse Effects of CBD

In the RCTs and their meta-analyses presented below, the adverse effects caused by CBD administered at daily dose ranges of 10–20 mg/kg [9], 5–50 mg/kg [10], 10–50 mg/kg [20], 5–25 mg/kg [12], and 5–20 mg/kg [21] were monitored. CBD was used as an adjunctive drug with a variety of antiepileptic medications.

In general, CBD treatment was related to a 1.12 times increase in the incidence of any grade of adverse effects, a 3.39 times increase in the incidence of severe-grade events, a 2.67 times rise in the occurrence of serious adverse effects, a 3.95 times increase in the incidence of undesired effects leading to treatment discontinuation, and a 9.87 times rise in the occurrence of events resulting in dose reduction [10,21]. The commonly observed significant vs. placebo adverse effects were the following: somnolence (*p* < 0.001), decreased appetite (*p* < 0.001), diarrhea (*p* = 0.001), and increased alanine or aspartate aminotransferases more than 3 times the upper normal limit (*p* < 0.001). Others did not reach the level of significance, like upper respiratory tract infections (*p* < 0.875), pyrexia (*p* = 0.681), vomiting 11.5% (*p* = 0.729), and sedation (*p* = 0.063) [9,10,12,20,21]. In addition, a dose–effect relationship was observed as undesired effects were more frequent and intense in the patients treated with higher doses of CBD [9,12,20]. More serious adverse events, like respiratory tract infections (pneumonia), liver failure, or status epilepticus, were also observed. It should be remembered, however, that some severe adverse events can be a result not only of the pharmacotherapy with CBD or co-administered antiepileptic drugs but also due to the severity of the patients’ general condition [20,21]. An important observation was that undesired effects, especially mild and moderate ones, resolved after dose reduction or withdrawal of CBD and/or the co-administered antiepileptic drug [9,10,12,20,21].

A meta-analysis by Chesney et al. [21] underlined that the incidences of abnormal liver function, somnolence, sedation, and pneumonia were examined only in the childhood epilepsy studies, where CBD could interact with medications such as clobazam and/or valproate. When such studies were excluded, the only adverse effect related to CBD treatment that remained was diarrhea. Similarly, other meta-analyses showed that more than two-thirds of the patients with elevated transaminases were co-treated with valproate. As CBD did not significantly increase the plasma concentrations of valproate, the interaction between the two drugs seems to be of a pharmacodynamic nature. In all the cases, the transaminase levels returned to normal values either spontaneously or after a reduction in the valproate dose. On the other hand, somnolence was the most frequent in patients co-treated with clobazam. A reduction in the clobazam dose decreased the frequency of this adverse effect. Nevertheless, the clobazam-off patients also experienced somnolence, especially those taking CBD at the dose of ≥20 mg/kg/day. Pneumonia and rash developed only in the patients co-treated with CBD and clobazam and were not enhanced with the increasing dose of CBD [6,7,8,9,26]. Among gastrointestinal adverse effects, diarrhea can be, at least partially, a consequence of the unbeneficial action of the sesame oil-based drug vehicle on the gut microbiome. Loss of appetite and decreased weight are probably directly related to the CBD treatment as they also occurred independently from diarrhea. Clinically significant weight loss emerged typically only after about 6 months of therapy. Furthermore, a single-center systematic review reported thrombocytopenia in 10% of the pediatric and adolescent patients diagnosed with LGS, DS, or other DREs who were treated concomitantly with CBD and valproate. Thrombocytopenia resolved after reducing the CBD and/or VPA doses [9].

Therefore, it can be assumed that CBD is well-tolerated and produces relatively few serious adverse effects. However, the pharmacokinetic interactions of CBD with other antiepileptic drugs deserve to be emphasized. As mentioned earlier, CBD is a potent inhibitor of CYP3A4 and CYP2C19 cytochromes. These hepatic enzymes metabolize both clobazam and valproate. The inhibition of CYP2C19 can increase the levels of N-CLB 2-7-fold, an active metabolite of clobazam, which translates into a significant sedative effect. In experimental conditions, very high acute doses of CBD (1.5 and 4.5 g orally) had limited effects on vigilance, suggesting that CBD at such doses does not produce a sedative effect. Interestingly, elevated N-CLB levels, through increasing the risk of sedation, respiratory depression, and aspiration, can account for the enhanced odds of pneumonia in patients taking CBD at a dose above 10 mg/kg/day. Abnormal liver function tests were observed mostly in children with epilepsy treated with valproate. The exact mechanism of this interaction is not fully clear. The co-administration of CBD and valproate did not significantly alter the plasma levels of their metabolites. However, 7-COOH-CBD, valproate, and its metabolite 4-ene-valproic acid can mutually potentiate each other’s hepatotoxic effects. Finally, an increased risk of diarrhea and reduced appetite may result from the direct CBD effects on the ECS, one of the functions of which is the regulation of peristalsis and appetite [21].

## 7. Discussion

Numerous RCTs and their meta-analyses have indicated that CBD, a non-psychoactive component of *Cannabis sativa*, exhibits essential anticonvulsant properties, reducing the seizure frequency in patients with DRE. Promising results were reported primarily in children and adolescents with LGS, DS, and TSC as these age groups and these epilepsy syndromes have undergone the most research. However, positive outcomes have also been observed in Sturge–Weber syndrome, focal cortical dysplasia, lissencephaly, brain tumor-related epilepsy, frontal and temporal lobe epilepsy, and some rare forms of epilepsy, including CDKL5 deficiency disorder, SYNGAP1 encephalopathy, epilepsy with myoclonic absences, as well as Aicardi, Dup15q, and Doose syndromes. Notably, a dose–effect relationship has been observed in relation to both the therapeutic and side effects induced by CBD. In this regard, the optimal dose of the cannabinoid seems to be 20 mg/kg/day. It may be problematic that CBD and other cannabinoids have complex and not entirely discovered mechanisms of action, involving effects on various channels and receptors, including GABA and NDMA. This means that CBD can interact with antiepileptic drugs, with which they share their pathways, particularly in patients with complex medical conditions or those on multiple medications. Actually, this refers to most cases of DREs. For ethical reasons, research on CBD is always carried out on patients concomitantly treated with antiepileptic therapy. So, drug–drug interactions (both pharmacokinetic and pharmacodynamic) may greatly contribute to either therapeutic or adverse effects of CBD. Nevertheless, the antiseizure action of the cannabinoid was indirectly confirmed in experimental studies, and directly proved in clinical conditions. As previously mentioned, CBD elevates the plasma concentrations of N-CLB by about three times the active metabolite of clobazam. Reciprocally, clobazam raises the plasma levels of 7-hydroxy-CBD, the active metabolite of CBD, by around 1.5 times. However, in patients concomitantly treated with stiripentol, the N-CLB levels did not rise as the stiripentol already saturated the binding sites within the CYP2C19 cytochrome. Therefore, an increase in N-CLB plasma concentrations is not required to achieve the antiseizure activity of CBD. A pharmacodynamic synergistic interaction between CBD and clobazam is also possible [26,28,30].

CBD is considered to be a safe medication. The most common adverse effects include diarrhea, decreased appetite, somnolence, and increased levels of hepatic aminotransferases. It is worth mentioning that the majority of patients with elevated aminotransferases were co-treated with valproate. Furthermore, concomitant treatment with the two medications led to thrombocytopenia in 10% of the patients. Noteworthily, the interaction between CBD and valproate seems to be of a pharmacodynamic nature. On the other hand, somnolence was the most frequent in those patients co-administered clobazam. Nevertheless, the clobazam-off patients also experienced somnolence, especially those taking CBD at the dose of ≥20 mg/kg/per day. In addition, pneumonia and rash developed only in the patients on CBD and clobazam, and they did not worsen with increased doses of CBD. Importantly, in all the cases of polytherapy, the adverse effects were reduced or even abolished after the dose reduction of the respective antiepileptic drug. On the other hand, loss of appetite and decreased weight seem to be directly related to CBD [6,7,8,9,10,28].

Studies on CBD still present numerous limitations. The authors of meta-analyses often underscore the availability of too few RCTs and insufficient group sizes. Also, the ethnic diversity of the patients was low, more than 90% of the participants being Caucasian, which makes it impossible to generalize the conclusions to the entire population. The effects of CBD, applied at the dose of 10 mg/kg/day, on different seizure parameters and safety profiles have too seldom been studied. Therefore, an exact dose–effect relationship cannot be properly evaluated. Due to the limited treatment and follow-up times, conclusions regarding long-term therapy cannot be drawn. The concomitant use of several antiepileptic drugs, which can interact with CBD, makes it impossible to determine the effectiveness of CBD alone and in comparison with co-administered antiseizure medications. A limitation of the trials evaluating the concomitant treatment with CBD and clobazam is that the clobazam-off groups were recruited from patients who previously did not respond to this antiepileptic drug. The comparative analysis of the drugs used in the treatment of LGS did not include studies where steroids or ACTH therapy were used, while these drugs are some of the most important in the treatment of this syndrome [28]. Most of the studies were conducted in children and adolescents with LGS and DS. Therefore, not much can be said about CBD treatment in adults and in other types of epilepsy. Furthermore, there is insufficient data regarding the age- and sex differences in clinical response, efficacy, and safety profile related to the treatment with CBD. The study design often excludes patients who previously experienced seizure worsening, which is a frequently observed phenomenon in children with epileptic encephalopathies and refractory seizures. In the case of non-convulsive seizures, which are not easy to observe by caregivers in developmentally delayed children, it is difficult to draw reliable conclusions [6,7,10]. Finally, sponsor-related bias should not be underestimated. Some RCTs and meta-analyses were conducted by the same research group, sometimes with links to the manufacturer. Finally, further intense research is necessary to achieve a complete understanding of the CBD mechanisms of action and its interactions with other drugs [20].

## 8. Conclusions

The results of 13 meta-analyses, including 225 RCTs presented herein, confirm the effectiveness of CBD in children and adolescents with DREs. Moreover, the safety level of CBD use seems to be satisfactory. The adverse effects reported by patients were usually transient and of mild to moderate intensity. In many cases, the side effects appeared during polytherapy with antiepileptic drugs, particularly clobazam and valproate. However, in all the described cases, such negative consequences of the combined treatment were reduced or abolished after reducing the dose of the antiepileptic medication. CBD’s complex mechanism of action predisposes it to interact with other drugs. The most-studied are those with clobazam and valproate. Ongoing research should shed more light on CBD’s interactions with other medications, the optimal dosing, and long-term therapeutic effects, as well as its potential use in adult patients and beyond the reported epilepsy syndromes.

## Figures and Tables

**Table 1:** Brief comparison of meta-analyses assessing the effectiveness of CBD in selected epilepsy syndromes.

Number of RCTs and (Participants)	Epilepsy Syndrome	Daily Dose of CBD	Effect of CBD at the Highest Dose on SFR (*p*-Value)	Effectiveness of CBD in CLB-on vs. CLB-off Groups	Reference
4 (550)	LGS, DS	10, 20 mg/kg for up to 14 weeks	*p* = 0.001	na	[6]
3 (359)	DS	10, 20 mg/kg for up to 14 weeks	*p* = 0.001	CLB-on > CLB-off	[7]
4 (714)	LGS, DS	10, 20 mg/kg for 14 weeks	*p* = 0.019	CLB-on = CLB-off	[8]
4 (716)	LGD, DS	10, 20 mg/kg for 14 weeks	*p* = 0.0226	CLB-on = CLB-off	[28]
4 (716)	LGS, DS	10, 20 mg/kg for 14 weeks	*p* < 0.0001	CLB-on = CLB-off	[26]
na (224)	TSC	25, 50 mg/kg for up to 16 weeks	*p* = 0.002	na	[9]
6 (972)	LGS, DS. TSC	5–50 mg/kg for up to 18 weeks	*p* < 0.01	CLB-on > CLB-off	[20]
152 (12, 123)	unspecified epilepsy	10–20 mg/kg for up to 18 weeks	*p* < 0.00001	CLB-on = CLB-off	[29]
9 (202)	LGS	20 mg/kg for up to 15 weeks	nd	na	[30]

CBD, cannabidiol; RCT, randomized clinical trial; CLB, clobazam; LGS, Lennox–Gastaut syndrome; DS, Dravet syndrome; TS, tuberous sclerosis complex.

## Data Availability

The data presented in this study are available on request from the corresponding author.
